# The mediating effect of anger rumination, coping and conformity motives on the association between hostility and problematic cannabis use

**DOI:** 10.1016/j.abrep.2022.100447

**Published:** 2022-07-09

**Authors:** Zsolt Horváth, Gyöngyi Kökönyei, Péter Sárosi, Mónika Koós, Zsolt Demetrovics, Róbert Urbán

**Affiliations:** a Institute of Psychology, ELTE Eötvös Loránd University, Hungary; b SE-NAP2 Genetic Brain Imaging Migraine Research Group, Hungarian Academy of Sciences, Semmelweis University, Budapest, Hungary; c Department of Pharmacodynamics, Faculty of Pharmacy, Semmelweis University, Budapest, Hungary; d Doctoral School of Psychology, ELTE Eötvös Loránd University, Hungary; e Centre of Excellence in Responsible Gaming, University of Gibraltar, Gibraltar

**Keywords:** Anger rumination, Hostility, Cannabis use, Coping motives, Conformity motives

## Abstract

**Background:**

Anger rumination is consistently associated with maladaptive psychopathological outcomes. However, there is a lack of research on the association between problematic cannabis use, cannabis use motives and anger rumination. Coping motives showed positive relationships with negative affectivity and emotion dysregulation, thus it might be possible that coping motives can mediate the effects of hostility and anger rumination on problematic cannabis use.

**Aims:**

The goal of the present study was to examine the mediating role of anger rumination and cannabis use motives on the relationship between hostility and problematic cannabis use.

**Methods:**

The cross-sectional study used a convenience sample of 764 past year cannabis users with a risk for problematic cannabis use (Males: 70.42% [N = 538], Age: M = 29.24 [SD = 7.55]). Standardized and online questionnaires measured problematic use and motives of cannabis use, anger rumination and hostility.

**Results:**

Structural equation modelling was used to test the proposed mediation model. Four significant indirect effects were identified in the mediation model. The positive effect of hostility on cannabis use problems was mediated (i) via coping motives, (ii) via anger rumination, (iii) via anger rumination and coping motives, and (iv) via anger rumination and conformity motives.

**Conclusions:**

The present study was the first that examined the construct of anger rumination in the context of cannabis use. Possible risk mechanisms via anger rumination and cannabis use motives with negative reinforcement were suggested in the context of problematic cannabis use. Self-medication tendencies and emotion dysregulation processes might explain these pathways.

## Highlights

• The role of anger rumination on cannabis use was examined for the first time.
• Indirect effects were tested between hostility and cannabis use problems.
• Single-mediation pathways were shown via anger rumination, and coping motives.
• Double mediation pathways were shown via anger rumination and coping-, and conformity motives.

## 1 Introduction

### 1.1 Problematic cannabis use

According to the 11th revision of the International Classification of Diseases, different forms of problematic cannabis use can form a hierarchical structure (Saunders, 2017, World Health Organization, 2018). In its most severe form, cannabis dependence is characterized by symptoms of impaired control over cannabis use, the central role and priority of cannabis use in one’s life despite the presence of negative consequences, craving, withdrawal symptoms and tolerance related to cannabis use (World Health Organization, 2018). Harmful cannabis use describes a problematic pattern of cannabis use (in the absence of cannabis dependence) that can lead to significant harm to the individual’s life (e.g., using cannabis in a hazardous way, cannabis use leading to legal, social or interpersonal problems or failures to fulfil role obligations) or to others (e.g., family members) (Lago et al., 2016, World Health Organization, 2018). In addition to these, it is worth to mention the category of hazardous cannabis use as well. This pattern of cannabis use can increase the risk of the development of harmful consequences in the individual’s and others’ life due to risky and non-standard cannabis use characteristics (e.g., frequency and quantity of cannabis use, context of use, risky behaviors related to cannabis use), though it has not led yet to negative consequences (World Health Organization, 2018). According to the representative data of the World Mental Health Survey, 9% and 3% of the cannabis users show harmful use and dependence, respectively (Degenhardt et al., 2018).

### 1.2 The relationship between anger/hostility and problematic cannabis use

Problematic cannabis use is positively associated with various dimensions of negative affectivity, such as major depressive disorder and generalized anxiety disorder (Lev-Ran et al., 2014, Onaemo et al., 2021). Among negative affective states, it is important to highlight the role of anger/hostility. In the present study we define anger/hostility primarily as a constellation of distressful affective symptoms and behaviors (e.g., irritability, aggressive urges and behaviors) (Urbán et al., 2014). However, it is important to note that other conceptualizations of the constructs of anger and hostility are also reported in the literature (e.g., hostility is primarily characterized by a set of negative attitudes and cognitions towards others [for example: devaluation, cynicism and mistrust], anger covers wide range of physiological, emotional, cognitive and behavioral characteristics [for example: intense arousal, irrational beliefs, verbal and behavioral expression of anger]) (Eckhardt et al., 2004). Similar to the present paper, several previous studies viewed anger and hostility as interchangeable constructs, but it is also assumed that “the term hostility is more specifically reserved for frequently recurring anger or anger proneness” (Fernandez et al., 2015, p. 74).

A complex and bidirectional relationship is suggested between anger/hostility and cannabis use. Numerous previous studies showed positive associations between cannabis use and anger/hostility (Ansell et al., 2015, Dillon et al., 2021, Pahl et al., 2011, Wycoff et al., 2018). However, it was also reported that cannabis use can lead to reductions in the level of anger/hostility (Wycoff et al., 2018). Multiple mechanisms were assumed which can account for the positive link between anger/hostility and cannabis use. For example, emotional states and thoughts related to anger/hostility can emerge as a result of cannabis withdrawal (Allsop et al., 2011). In line with this, it is also possible that one might use cannabis to cope with anger/hostility. In addition to these, intensive cannabis use can also lead to higher levels of anger/hostility, for example, via hostile interpersonal cognitions and reactions (Ansell et al., 2015, Dillon et al., 2021).

### 1.3 Anger rumination

Emotion regulation processes related to anger/hostility can also provide an explanation for the positive link between cannabis use and anger/hostility. It is important to examine the role of anger rumination (AR) in this regard. As a subtype of repetitive negative thinking (RNT), it is defined as a preference for perseverative and reoccurring thinking related to anger episodes (Ciesla et al., 2011, Denson, 2013, Sukhodolsky et al., 2001). AR is a multidimensional construct characterizing an emotion regulation process that includes concentrating on anger-related emotional states, re-experiencing anger events, having thoughts about the causes and consequences of anger events as well as thinking about revenge (Sukhodolsky et al., 2001). AR can be a risk factor for various maladaptive psychopathological outcomes. Higher levels of AR can maintain and intensify the level of anger/hostility (Denson, 2013). Moreover, previous research showed that AR is positively correlated with transdiagnostic factors of internalizing and externalizing psychopathologies, symptoms of antisocial and borderline personality disorder, and various dysregulated behaviors (e.g., aggressive behaviors) (du Pont et al., 2018, du Pont et al., 2019, Kelley et al., 2021, Peled and Moretti, 2010).

Among dysregulated behaviors, less research has focused specifically on the relationship between AR and problematic substance use. The existing findings predominantly investigated the link between alcohol use and AR. For example, previous studies demonstrated a positive relationship between AR and frequency of alcohol use as well as a positive link between alcohol use and aggression among those with higher levels of AR (Borders and Giancola, 2011, Ciesla et al., 2011). However, there is a lack of research which examined the specific association between problematic cannabis use and AR. Based on the beforementioned research findings on the positive correlations between externalizing behaviors and AR, it might be worth to assume that AR can also associated with more problematic cannabis use.

Although there is a dearth of research on the specific association between problematic cannabis use and AR, the relationship between RNT and cannabis use is well documented. Namely, higher levels of cannabis use-related problems are positively associated with specific subtypes of depressive rumination, such as of brooding and problem-focused thinking, and with post-event processing (Adrian et al., 2014, Bravo et al., 2019, Ecker and Buckner, 2018). Moreover, these findings also revealed that RNT mediates the associations between psychopathological symptoms (e.g., depression, social anxiety) and problematic cannabis use. That is, higher levels of depression and social anxiety can lead to increased rates of brooding and post-event processing, respectively, which in turn can contribute to more problematic cannabis use (Adrian et al., 2014, Ecker and Buckner, 2018). Building on the latter literature results, it might be plausible to assume a similar mediating effect in the context of anger/hostility and AR: a positive effect of anger/hostility on problematic cannabis use might be presented via increased AR.

Finally, the emotional cascade model (ECM) can also serve as a basis to explain the relationships between anger/hostility, AR and problematic cannabis use (Selby et al., 2008, Selby et al., 2009). The ECM assumes that negative psychopathological symptoms (e.g., depressive symptoms, anger/hostility) can facilitate using ruminative emotion regulation strategies. However, ruminative processes can maintain and intensify these negative affective symptoms, which in turn can further promote rumination. This bidirectional mechanism might motivate the affected individuals to rashly engage in dysregulated behaviors (e.g., substance use, self-harm, binge eating) as a means for coping with negative psychopathological symptoms and shifting away from distressful thoughts (Kelley et al., 2021, Selby et al., 2008, Selby et al., 2009). For example, the ECM can predict the following mechanism in the context of anger/hostility, AR and cannabis use. An individual might experience anger/hostility-related affective symptoms (e.g., irritability, distress, aggressive urges) due to an anger/hostility-provoking event. As a response to these distressful affective states, one might intensively start concentrating and thinking on the anger/hostility-provoking event (e.g., thinking about revenge, the causes and consequences of that event, re-experiencing the event). This might subsequently lead to the exacerbation of distressful emotional states related to anger/hostility. Therefore, an individual might use cannabis in order to alleviate the intensity of distressful affective and cognitive symptoms related to anger/hostility.

### 1.4 Cannabis use motives

In addition to AR, other constructs can also explain the relationship between anger/hostility and problematic cannabis use, such as cannabis use motives. In line with the alcohol motivation model, individuals use cannabis in order to attain a desirable or avoid an undesirable pharmacological or psychosocial outcome (Cooper et al., 2015). Existing studies identified five main motives of cannabis use (Cooper et al., 2015, Simons et al., 1998). In accordance with the alcohol motivation model, four motives of cannabis use can be discriminated based on the source and reinforcement of the motives: social (external source and positive reinforcement – e.g., using cannabis to make a social event more fun), enhancement (internal source and positive reinforcement – e.g., using cannabis to experience positive sensations), conformity (external source and negative reinforcement – e.g., using cannabis to avoid social disapproval), and coping motives (internal source and negative reinforcement – e.g., using cannabis to cope with depressive or anxious symptoms). In addition to these, expansion motives describe a tendency to use cannabis to enhance awareness, openness and creativity (Cooper et al., 2015, Simons et al., 1998). According to a meta-analytic study, higher frequency of cannabis use is associated with higher rates of coping, enhancement and expansion motives and lower levels of conformity motives, whereas more problematic cannabis use is positively linked to coping and conformity motives (Bresin & Mekawi, 2019).

The framework of substance use motives assumes that motives are the most proximal determinants of substance use behavior, thus they can mediate the effects of more distal antecedents (e.g., personality traits, emotional characteristics) on outcomes of substance use. Moreover, it is expected that different motives of substance use is correlated with distinct personality and emotional antecedents (Cooper et al., 2015, Kuntsche et al., 2005). In terms of cannabis use motives, coping motives consistently presented positive associations with internalizing symptoms (e.g., depression, general anxiety, social anxiety) and difficulties of emotion regulation (e.g., low distress tolerance, experiential avoidance) (Buckner et al., 2014, Bujarski et al., 2012, Cooper et al., 2015, Farris et al., 2016). Moreover, the mediating effect of coping motives was also demonstrated between problematic cannabis use and distressful affective symptoms, emotion regulation difficulties (Buckner et al., 2007, Bujarski et al., 2012, Farris et al., 2016). However, very few literature data are available regarding the relationship between RNT and cannabis use motives, whereas there is a lack of research which examined the associations between cannabis use motives and AR. In a large, multinational study, a complex double-mediation model was tested on the relationship between depressive symptoms and cannabis use outcomes via the sequential mediating effects of depressive rumination and cannabis use motives (i.e., the latter being more proximal predictor on the outcome variable) (Bravo et al., 2019). Double-mediated effects suggested that higher levels of depressive symptoms are associated with problem-focused thoughts which in turn are related to higher rates of coping motives which subsequently had positive effects on higher cannabis use and negative consequences (Bravo et al., 2019).

Considering these previous findings, it might be plausible to hypothesize that coping motives can be associated with increased levels of anger/hostility and AR – as these constructs can also represent distressful and negative affective states and maladaptive emotion regulation strategies. Moreover, based on the assumptions of the substance use motivation model and the findings of Bravo et al. (2019), it can be assumed that coping motives are more proximal determinants of problematic cannabis use than anger/hostility and AR, thus it might be possible that coping motives mediate the effects of anger/hostility and AR on problematic cannabis use.

### 1.5 Hypothesized mediational model

**Fig. 1:**
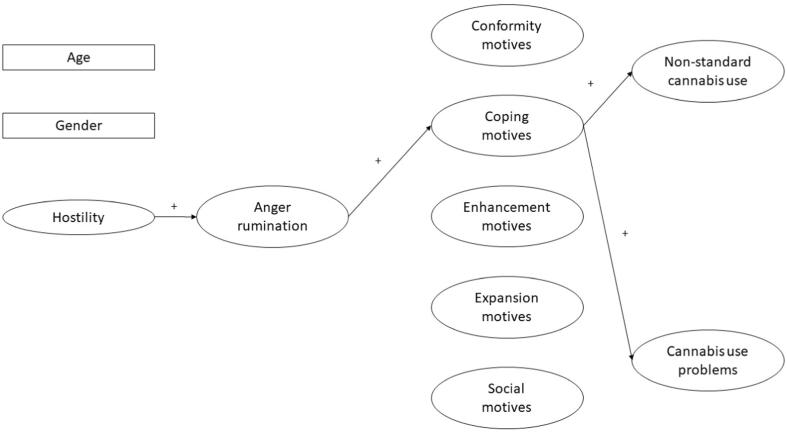
The hypothesized mediating effect of anger rumination and coping motives between hostility and problematic cannabis use. Positive signs indicate the assumed positive associations between the variables. The effect of age and gender was controlled in the analyses. In the complete mediation model all regression effects and indirect effects were estimated (not shown in this figure to ease the interpretation).

The present study aimed to examine how the constructs of AR and cannabis use motives can account for the relationship between hostility and problematic cannabis use. Specifically, the mediating role of AR and cannabis use motives on the relationships between hostility and non-standard cannabis use and cannabis use problems were tested. The hypothesized conceptual model of the study is shown in Fig. 1. The present study assumed that higher levels of hostility can be associated with elevated rates of AR which in turn can predict more problematic cannabis use via increased rates of coping motives (over the effects of gender, age and other motivational dimensions of cannabis use). However, it is important to note that the cross-sectional nature of the study did not allow to explore the bidirectional associations between the variables of the mediation model. The theoretical assumptions of the substance use motivation model as well as previous empirical findings were considered on the decision of the order of the variables in the mediation model. First, the model of substance use motives assumes that motives are the most proximal determinants of substance use behavior and they can mediate the more distal effects of emotional characteristics on outcomes of substance use (Cooper et al., 2015). Second, previous cross-sectional studies which simultaneously examined the mediating effects of depressive rumination and motives of alcohol and cannabis use also hypothesized that the effects of depression on outcomes of alcohol and cannabis use are sequentially mediated by depressive rumination and motives – with the latter being more proximal predictor on the outcome variables (Bravo et al., 2018, Bravo et al., 2019). Therefore, to correspond with these literatures, it was decided to have hostility as a distal predictor variable, AR as a distal mediator variable and cannabis use motives as proximal mediator variables. As there is a lack of research which examined the complex associations between AR, cannabis use motives and problematic cannabis use, this approach might allow to make comparisons with Bravo et al.’s (2019) study which examined a similar research question but in the context of depressive rumination.

## 2 Methods

### 2.1 Participants and procedures

The present cross-sectional study used online questionnaires to assess psychological correlates of cannabis use (Horváth et al., 2022). Convenience sampling was performed among Hungarian, potential problematic cannabis user individuals. Invitation to participate in the study was posted in cannabis- and illicit drug use-related Facebook pages and groups. Participants provided informed consent before starting the completion of the online questionnaire. Participation in the study was voluntary, anonymous, and individuals did not receive any compensation for that. The research protocol was approved by the Research Ethics Committee at the Faculty of Education and Psychology of the ELTE Eötvös Loránd University, Budapest, Hungary (approval number: 2020/407).

Overall, 1359 individuals participated in the present study. However, data from male and female cannabis users who showed a risk for problematic cannabis use in the past 12 months with valid (non-missing) answers on all the relevant questionnaires of the present study were considered in the final analyses. The risk for problematic cannabis use was assessed by using the Cannabis Abuse Screening Test (CAST): only those participants were included in the final sample who had 3 or more points on the total scale score (Legleye et al., 2011). Thus, the final sample consisted of data from 764 respondents. Table 1 presents the sample characteristics. High proportions of the sample were male, had high school degree or at least college or university degree, lived in the capital city (Budapest) or in cities, worked in full-time, and did not study. In terms of cannabis use, most of the participants used it four or more times a week.

**Table 1:** Sample characteristics and descriptive statistics of the questionnaires (N = 764).

Gender N (%)	
Females	226 (29.58%)
Males	538 (70.42%)
Age M (SD)	29.24 (7.55)
Educational attainment N (%)	
Absence of secondary educational attainment	55 (7.20%)
Vocational school	139 (18.19%)
High school degree	232 (30.37%)
Technical training after secondary school	137 (17.93%)
College or university degree or higher	201 (26.31%)
Settlement type N (%)	
Capital city (Budapest)	297 (38.87%)
Cities	291 (38.09%)
Villages	128 (16.75%)
Foreign, non-Hungarian settlement	48 (6.28%)
Working status N (%)	
Working in full-time	519 (67.93%)
Working in part-time or occasionally	145 (18.98%)
Not working currently	100 (13.09%)
Status of current studies N (%)	
Currently studying	222 (29.06%)
Not studying currently	542 (70.94%)
Frequency of cannabis use in the past 12 months N (%)	
Monthly or less	61 (7.98%)
Two-four times a month	114 (14.92%)
Two-three times a week	147 (19.24%)
Four or more times a week	442 (57.85%)
Hostility M (SD)	3.57 (3.53)
Anger rumination M (SD)	34.01 (9.25)
Conformity motives M (SD)	5.74 (1.53)
Coping motives M (SD)	11.56 (4.74)
Enhancement motives M (SD)	15.83 (4.05)
Expansion motives M (SD)	14.91 (5.85)
Social motives M (SD)	11.72 (4.73)
Problematic cannabis use M (SD)	7.76 (3.12)

### 2.2 Measures

#### 2.2.1 Brief symptom Inventory (BSI) – Hostility

The Brief Symptom Inventory’s (BSI) 5-item Hostility subscale was used to measure the frequency of hostility in the past 7 days (Derogatis and Savitz, 2000, Unoka et al., 2004). Each symptom (e.g., temper outbursts, urges to harm someone or things) was rated on a 5-point scale (0 = Not at all, 4 = Extremely). High level of internal consistency was shown for the scale in the sample (ω = 0.87; Supplementary Table 1).

#### 2.2.2 Anger rumination scale (ARS)

The construct of AR was assessed by the 19-item Anger Rumination Scale (ARS) (Sukhodolsky et al., 2001, Szabó and Kökönyei, 2021). Participants provided responses on a 4-point scale for each item that covered experiences and thoughts related to anger/hostility (1 = Nearly never, 4 = Nearly always). The ARS measures four main dimensions of AR: (i) angry afterthoughts (AA; e.g., “After an argument is over, I keep fighting with this person in my imagination”), (ii) thoughts of revenge (TR; e.g., “I have long living fantasies of revenge after the conflict is over”), (iii) angry memories (AM; e.g., “I keep thinking about events that angered me for a long time”) and (iv) understanding causes (UC; e.g., “I analyze events that make me angry”). In an orthogonal bifactor measurement model of the ARS (Spyropoulou & Giovazolias, 2021), the general AR factor was characterized by high internal consistency in the sample (ωH = 0.90), whereas the specific AR factors had low specific internal consistencies if the effect of the general AR factor was controlled (AA: ωH = 0.13; AM: ωH = 0.08; TR: ωH = 0.32; UC: ωH = 0.21; Supplementary Table 2).

#### 2.2.3 Marijuana motives measure (MMM)

The motives for using cannabis in the past 12 months were measured by the 25-item Marijuana Motives Measure (MMM) (Simons et al., 1998). It covers four main dimensions of cannabis use motives: (i) conformity (e.g., “Because my friends pressure me to use marijuana”), (ii) coping (e.g., “Because it helps me when I feel depressed or nervous”), (iii) enhancement (e.g., “To get high”), (iv) expansion (e.g., “Because it helps me be more creative and original”) and (v) social motives (e.g., “Because it makes social gatherings more fun”). A 5-point scale was used to estimate the frequency of each motive for cannabis use (1 = Never/Nearly never, 5 = Nearly always/Always). Factors of cannabis use motives showed high-very high levels of internal consistency in the sample (conformity motives: ω = 0.86, coping motives: ω = 0.90, enhancement motives: ω = 0.83, expansion motives: ω = 0.93, social motives: ω = 0.88; Supplementary Table 4).

#### 2.2.4 Cannabis Abuse Screening test (CAST)

The 6-item Cannabis Abuse Screening Test (CAST) estimated the level of problematic cannabis use in the past 12 months (Gyepesi et al., 2014, Legleye et al., 2007). Participants rated the level of problems and risky, non-standard consumption forms related to cannabis use on a 5-point scale (0 = Never, 4 = Very often). In line with some of the previous, factor analytic findings (Cuenca-Royo et al., 2012, Legleye, 2018, Sznitman, 2017), two dimensions of problematic cannabis use were assessed by using the CAST: (i) non-standard cannabis use (i.e., cannabis use before midday and when alone), (ii) cannabis use problems (e.g., memory problems, unsuccessful attempts to reduce or stop cannabis use). Low internal consistencies were presented for both dimensions in the sample (non-standard cannabis use: ω = 0.53; cannabis use problems: ω = 0.63); however, items of the CAST showed moderate-high factor loadings on the two factors of problematic cannabis use (λ = 0.46–73; Supplementary Table 5).

### 2.3 Data analysis

All analyses were conducted by using the Mplus 8.0 statistical software (Muthén & Muthén, 2017). As preliminary analyses, means and standard deviations were calculated for the study questionnaires. Pairwise correlations were also calculated between the variables of the mediation model. McDonald’s omega (ω) was calculated for each scale to measure the level of internal reliability.

A multiple mediation model was specified by using structural equation modelling (SEM) to test the mediating role of AR and cannabis use motives on the relationships between hostility and non-standard cannabis use and cannabis use problems. Hostility was a distal predictor variable, AR and motives of cannabis use (conformity, coping, enhancement, expansion and social motives) were mediator variables at subsequent levels, and the main outcome variables were non-standard cannabis use and cannabis use problems. The effects of age and gender were also controlled in the mediation model (Fig. 1). Correlations between age, gender and hostility, between the five variables of cannabis use motives as well as between non-standard cannabis use and cannabis use problems were estimated. A two-step estimation method was applied. First, the complete mediation model was estimated. It covered all the beforementioned predictor, mediator and outcome variables, and all possible regression paths and indirect effects were estimated. Next, a trimmed mediation model was specified. Those variables were removed from the mediation model which did not contribute to significant indirect effects in the complete mediation model. This method can ensure to avoid increased rates of total explained variance by non-significant predictive effects.

Each variable in the model was specified as latent factors. Hostility was specified as a one-factor variable based on five observed indicators (i.e., items of the BSI Hostility subscale). Five correlating latent variables were specified to measure conformity, coping, enhancement, expansion and social motives. Each latent factor of cannabis use motives was defined based on five observed indicators (i.e., based on the corresponding items of each MMM subscale). A correlating two-factor structure represented problematic cannabis use: non-standard use was specified based on two observed indicators, whereas the latent factor of cannabis use problems was defined based on four observed indicators (i.e., the corresponding items of both CAST subscales). Originally, based on previous findings, it was aimed to specify a bifactor model to represent the latent construct of AR (Spyropoulou & Giovazolias, 2021). In an orthogonal bifactor model, each item of the ARS is simultaneously related to a higher-order, general AR factor and to one of the specific AR factors (AA, TR, AM, UC). The bifactor structure of the ARS can allow to compare the explained common variance between the general and specific AR factors. Factor loadings and internal reliability estimates in the bifactor model are shown in Supplementary Table 2. Except for one item, all ARS items had strong factor loadings on the general AR factor, and only two items presented stronger factor loadings on the specific AR factors than on the general AR factor. The general AR factor explained high proportion of the common variance and correlations in the model (ECV = 74%, PUC = 0.78; ω = 0.95; ωH = 0.90). Only small proportions of the variances were attributable to the specific AR factors on the weighted subscale scores if the effect of the general AR factor was controlled (ECV = 3–9%; ωH = 0.08–0.32). Only the general AR factor was characterized as a sufficiently defined latent factor (H > 0.80; FDI > 0.90). Overall, these measures indicated that the specific AR factors are not sufficiently defined latent constructs with low internal reliability and limited information capacity if the effect of the general AR factor is taken into account. Therefore, it was decided to remove the specific AR factors from the mediation model, and to specify the construct of AR as a one-factor variable in the mediation model. That is, each item of the ARS only loaded on a general AR factor (Supplementary Table 3). However, in order to model the specific associations between the items of each specific AR factor, error correlations were defined between AA-, AM-, TR-, and UC-specific items. This measurement model allowed that only the general AR factor was included in the mediation model (as a well-defined latent construct), but the specific associations between the items of the specific ARS subscales were also not disregard (though specific AR factors were not defined in the mediation model).

Multiple assumptions of SEM were considered prior the analyses (Kline, 2016). The complete and trimmed mediation models had degrees of freedom greater than zero. The scale of the latent variables was specified by setting the variance of each latent variable at 1. Thus, all factor loadings of the latent variables were estimated. Each latent variable in the mediation models was defined by at least 2 observed indicators. Multiple observed indicators showed non-normal distribution. Therefore, observed indicators of the latent factors were specified as ordered categorical indicators. The SEM parameters were calculated by using the weighted least squares means and variances adjusted (WLSMV) estimation method. The use of ordinal observed indicators and the WLSMV estimation (instead of continuous indicators and maximum likelihood-based estimation) can be preferable with non-normally distributed indicators (Li, 2016). During the model estimation processes, all parameters were identified and calculated, there were no convergence issues. It was intended to use the full information maximum likelihood (FIML) missing data handling method. However, all questions in the online questionnaire were mandatory and it was required from the participants in the final sample to have valid (non-missing) answers on all the relevant questionnaires of the present study. Thus, there were no missing observations on the variables of the mediation models. Extreme multicollinearity was not demonstrated between the variables (Table 2). At least moderately strong and significant factor loadings were presented for all latent factors in the mediation models which might indicate that the measurement models of the SEM were defined sufficiently (Supplementary Tables 1, and 3-5). Except for the latent factor of AR (i.e., error correlations were defined between the items of the specific AR factors), the assumption of local independence was met for the other latent factors and error correlations were not allowed between the observed indicators. Correlations were allowed between the latent variables at the same conceptual levels (i.e., between age, gender and hostility; between cannabis use motives; and between non-standard cannabis use and cannabis use problems). Multivariate outlier testing was not available for the mediation models because of the WLSMV estimation method.

**Table 2:** Pairwise correlations between the latent variables.

	1.	2.	3.	4.	5.	6.	7.	8.	9.	10.
1. Age	–									
2. Gender^1^	0.04	–								
3. Hostility	−0.05	−0.06	–							
4. Anger rumination	−0.11**	−0.10*	0.63***	–						
5. Conformity motives	−0.14**	0.06	0.14*	0.23***	–					
6. Coping motives	−0.18***	−0.08*	0.28***	0.29***	0.24***	–				
7. Enhancement motives	−0.18***	0.06	0.05	0.06	0.17**	0.48***	–			
8. Expansion motives	−0.13***	0.04	−0.09*	−0.01	0.06	0.28***	0.36***	–		
9. Social motives	−0.22***	0.09*	0.05	0.11**	0.46***	0.42***	0.62***	0.38***	–	
10. Non-standard cannabis use	0.05	0.12*	0.05	−0.10	−0.18**	0.33***	0.29***	0.23***	0.17**	–
11. Cannabis use problems	−0.08	0.05	0.37***	0.39***	0.37***	0.31***	0.09	−0.04	0.19***	0.19**

Note. N = 764. Level of significance: *p < 0.050; **p < 0.010; ***p < 0.001. ^1^Coded as: 0 = Females, 1 = Males.

The model fit of the multiple mediation models was rated on the comparative fit index (CFI), on the Tucker-Lewis index (TLI) and on the root mean square error of approximation (RMSEA). Adequate levels of model fit were indicated by values ≥ 0.900 on the CFI and the TLI and by values ≤ 0.080 on the RMSEA. Optimal levels of model fit were shown by values ≥ 0.950 on the CFI and the TLI and by values ≤ 0.050 on the RMSEA (Brown, 2015).

## 3 Results

### 3.1 Preliminary analyses

Table 1 shows means and standard deviations on the study questionnaires. Table 2 presents the pairwise correlations between the latent variables of the multiple mediation model. Hostility presented a significant, positive and strong correlation with AR (r = 0.63; p < 0.001); and a significant, positive and moderate correlation with cannabis use problems (r = 0.37; p < 0.001); and significant, positive and weak correlations with conformity (r = 0.14; p = 0.014) and coping motives (r = 0.28; p < 0.001). Higher levels of AR were significantly and weakly associated with lower age (r = -0.11; p = 0.006), female gender (r = -0.10; p = 0.011), higher rates of conformity (r = 0.23; p < 0.001), coping (r = 0.29; p < 0.001) and social motives (r = 0.11; p = 0.009); and were significantly and moderately associated with higher levels of cannabis use problems (r = 0.39; p < 0.001). Higher levels of conformity motives were significantly and weakly associated with lower age (r = -0.14; p = 0.006) and non-standard cannabis use (r = -0.18; p = 0.008), higher rates of coping (r = 0.24; p < 0.001) and enhancement motives (r = 0.17; p = 0.005); and were significantly, positively and moderately correlated with social motives (r = 0.46; p < 0.001) and cannabis use problems (r = 0.37; p < 0.001). Coping motives had significant, positive and weak relationships with lower age (r = -0.18; p < 0.001), female gender (r = -0.08; p = 0.040) and expansion motives (r = 0.28; p < 0.001); and significant, positive and moderate correlations with enhancement (r = 0.48; p < 0.001) and social motives (r = 0.42; p < 0.001), non-standard cannabis use (r = 0.33; p < 0.001) and cannabis use problems (r = 0.31; p < 0.001). Enhancement motives presented significant and weak relationships with lower age (r = -0.18; p < 0.001) and higher non-standard cannabis use (r = 0.29; p < 0.001); and a significant, positive and moderate correlation with expansion motives (r = 0.36; p < 0.001); and a significant, positive and strong association with social motives (r = 0.62; p < 0.001). Higher levels of expansion motives were significantly and weakly associated with lower age (r = -0.13; p < 0.001) and hostility (r = -0.09; p = 0.027) and with higher non-standard cannabis use (r = 0.23; p < 0.001); and had a significant, positive and moderate correlation with social motives (r = 0.38; p < 0.001). Social motives were significantly and weakly associated with lower age (r = -0.22; p < 0.001), male gender (r = 0.09; p = 0.017), and higher non-standard cannabis use (r = 0.17; p = 0.001) and cannabis use problems (r = 0.19; p < 0.001). Non-standard cannabis use showed significant, positive and weak correlations with male gender (r = 0.12; p = 0.013) and cannabis use problems (r = 0.19; p = 0.001).

### 3.2 Complete mediation model

The complete mediation model showed adequate-optimal degrees of model fit (χ^2^[1449] = 3737.54; p < 0.001; RMSEA [90% CI] = 0.045 [0.044–0.047]; CFI = 0.925; TLI = 0.918). Measurement models of the SEM are presented in Supplementary Table 1 and Supplementary Tables 3-5. All factor loadings were strong (λ > 0.50) and significant (p < 0.001) in the measurement models of hostility (Supplementary Table 1) and cannabis use motives (Supplementary Table 4). In the measurement models of AR (Supplementary Table 3), non-standard cannabis use, and cannabis use problems (Supplementary Table 5), factor loadings were strong (λ > 0.50) and significant (p < 0.001) – except for one observed indicator in each case which presented a moderately strong (λ > 0.30) factor loading. Predictive effects in the complete mediation model are summarized in Table 3. Higher levels of AR were associated with increased levels of hostility (with strong effect size) and with lower age (with weak effect size). AR had significant, positive and weak predictive effects on conformity and coping motives. Higher levels of hostility were significantly and weakly associated with higher rates of coping motives and lower rates of expansion motives. Significant, negative and weak relationships were shown between age and all five cannabis use motives. Male gender was significantly and weakly associated with higher levels of enhancement and social motives. Non-standard cannabis use was significantly and weakly linked to male gender and lower levels of AR and conformity motives, in addition to the significant, positive and moderate association with coping motives. Cannabis use problems presented significant, positive and weak associations with hostility, AR, conformity and coping motives.

**Table 3:** Predictive effects in the complete mediation model.

	Outcome variables							
	Anger rumination β (S.E.)	Conformity motives β (S.E.)	Coping motives β (S.E.)	Enhancement motives β (S.E.)	Expansion motives β (S.E.)	Social motives β (S.E.)	Non-standard cannabis use β (S.E.)	Cannabis use problems β (S.E.)
Age	−0.08 (0.04)*	−0.12 (0.05)*	−0.15 (0.04)***	−0.18 (0.04)***	−0.13 (0.04)***	−0.21 (0.04)***	0.09 (0.05)	0.00 (0.05)
Male gender (vs. female gender)	−0.06 (0.03)	0.09 (0.05)	−0.05 (0.04)	0.07 (0.04)*	0.04 (0.04)	0.11 (0.04)**	0.13 (0.05)**	0.08 (0.04)
Hostility	0.62 (0.03)***	−0.01 (0.09)	0.16 (0.06)**	0.03 (0.06)	−0.14 (0.06)*	−0.02 (0.06)	0.14 (0.08)	0.17 (0.07)*
Anger rumination	–	0.23 (0.09)*	0.17 (0.05)**	0.03 (0.06)	0.07 (0.06)	0.11 (0.06)	−0.21 (0.08)**	0.18 (0.07)*
Conformity motives	–	–	–	–	–	–	−0.29 (0.08)***	0.26 (0.08)**
Coping motives	–	–	–	–	–	–	0.34 (0.06)***	0.19 (0.06)**
Enhancement motives	–	–	–	–	–	–	0.10 (0.08)	−0.05 (0.07)
Expansion motives	–	–	–	–	–	–	0.10 (0.06)	−0.08 (0.06)
Social motives	–	–	–	–	–	–	0.09 (0.10)	0.01 (0.09)
Explained variance (R^2^)	40%	8%	12%	4%	3%	7%	28%	30%

Note. N = 764. Standardized regression coefficients (β) and the corresponding standard error (S.E.) values represent each predictive effect in the model. Level of significance: *p < 0.050; **p < 0.010; ***p < 0.001. Correlation between non-standard cannabis use and cannabis use problems were estimated in the model: r = 0.29 (p < 0.001). Correlations between the cannabis motives factors were estimated in the model. Range of correlations (r): 0.04 (correlation between conformity and expansion motives) – 0.60 (correlation between enhancement and social motives); r_Mean_ = 0.33. Except for the correlation between conformity and expansion motives, all correlations between cannabis use motives were significant (p < 0.050). Correlation estimates between cannabis use motives are presented in Supplementary Table 4. Correlations between hostility, age and gender were also estimated. All correlations between these variables were weak (|r|=0.04–0.07) and non significant (p ≥ 0.050).

Table 4 summarizes the total, direct and indirect effects between hostility and cannabis use outcomes. Hostility presented non-significant total and direct effects on non-standard cannabis use. Indirect effects between hostility and non-standard cannabis use were not estimated due to the non-significant total effect. Hostility presented significant total and direct effects on cannabis use problems. Additionally, four significant indirect pathways were identified in the complete mediation model. First, higher levels of hostility were associated with increased rates of AR which in turn had a positive effect on cannabis use problems. Second, higher levels of hostility predicted higher levels of coping motives which subsequently had a positive link with cannabis use problems. Third, hostility was positively related to elevated rates of AR which in turn predicted higher levels of conformity motives which in turn had a positive association with cannabis use problems. Fourth, hostility positively predicted AR which in turn was positively associated with coping motives which subsequently contributed to higher rates of cannabis use problems. These four indirect effects represented partial mediation effects due to the significant direct effect of hostility on cannabis use problems.

**Table 4:** Total, direct and indirect effects of hostility on cannabis use-related outcomes.

	Complete mediation model		Trimmed mediation model	
	Outcome: Non-standard cannabis use β (S.E.)	Outcome: Cannabis use problems β (S.E.)	Outcome: Non-standard cannabis use β (S.E.)	Outcome: Cannabis use problems β (S.E.)
Total effect	0.057 (0.055)	0.375 (0.048)***	0.050 (0.057)	0.373 (0.049)***
Direct effect	0.141 (0.078)	0.172 (0.070)*	0.116 (0.079)	0.187 (0.069)**
Total indirect effect	-^1^	0.203 (0.047)***	-^1^	0.186 (0.047)***
Hostility **→** Anger rumination **→** Cannabis use outcome	-^1^	0.111 (0.043)*	-^1^	0.109 (0.044)*
Hostility **→** Conformity motives **→** Cannabis use outcome	-^1^	−0.001 (0.024)	-^1^	−0.002 (0.025)
Hostility **→** Coping motives **→** Cannabis use outcome	-^1^	0.030 (0.014)*	-^1^	0.021 (0.011)*
Hostility **→** Enhancement motives **→** Cannabis use outcome	-^1^	−0.002 (0.004)	-^2^	-^2^
Hostility **→** Expansion motives **→** Cannabis use outcome	-^1^	0.011 (0.009)	-^2^	-^2^
Hostility **→** Social motives **→** Cannabis use outcome	-^1^	0.000 (0.002)	-^2^	-^2^
Hostility **→** Anger rumination **→** Conformity motives **→** Cannabis use outcome	-^1^	0.037 (0.019)*	-^1^	0.041 (0.018)*
Hostility **→** Anger rumination **→** Coping motives **→** Cannabis use outcome	-^1^	0.020 (0.010)*	-^1^	0.017 (0.008)*
Hostility **→** Anger rumination **→** Enhancement motives **→** Cannabis use outcome	-^1^	−0.001 (0.002)	-^2^	-^2^
Hostility **→** Anger rumination **→** Expansion motives **→** Cannabis use outcome	-^1^	−0.003 (0.004)	-^2^	-^2^
Hostility **→** Anger rumination **→** Social motives **→** Cannabis use outcome	-^1^	0.001 (0.006)	-^2^	-^2^

Note. β (S.E.): Standardized effect size with the related standard error value. Level of significance: *p < 0.050; **p < 0.010; ***p < 0.001. ^1^The indirect effects between hostility and non-standard cannabis use were not estimated due to the non-significant total effect between the variables. ^2^The indirect effects were not included in the trimmed mediation model.

### 3.3 Trimmed mediation model

**Fig. 2:**
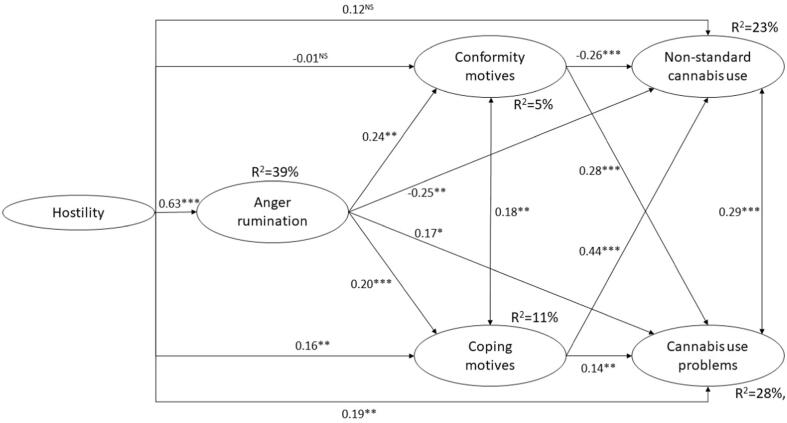
Significant predictive effects in the trimmed mediation model. N = 764. Single-headed arrows are regression predictive effects and values in these arrows are standardized regression coefficients (β). Double-headed arrows are correlations between the latent variables (r). Level of significance: ^NS^p ≥ 0.050; *p < 0.050; **p < 0.010; ***p < 0.001.

Next, a trimmed mediation model was specified. Age, gender, enhancement, expansion and social motives were removed from the mediation model as these variables did not contribute to significant indirect effects in the complete mediation model. Therefore, hostility, AR, conformity and coping motives, non-standard cannabis use, and cannabis use problems were included in the trimmed mediation model. Optimal levels of model fit were shown for this mediation model (χ^2^[6 8 8] = 1462.88; p < 0.001; RMSEA [90% CI] = 0.038 [0.036–0.041]; CFI = 0.961; TLI = 0.956). Measurement models of the SEM are presented in Supplementary Table 1 and Supplementary Tables 3-5. All factor loadings were strong (λ > 0.50) and significant (p < 0.001) in the measurement models of hostility (Supplementary Table 1), conformity motives (Supplementary Table 4), and non-standard cannabis use (Supplementary Table 5). In the measurement models of AR (Supplementary Table 3), coping motives (Supplementary Table 4) and cannabis use problems (Supplementary Table 5), factor loadings were strong (λ > 0.50) and significant (p < 0.001) – except for one observed indicator in each case which presented a moderately strong (λ > 0.30) factor loading. Predictive effects in the trimmed mediation model are shown in Fig. 2. Hostility had significant and positive predictive effects on AR (with strong effect size), coping motives and cannabis use problems (with weak effect sizes). Higher levels of AR were significantly and weakly linked to elevated rates of conformity and coping motives and cannabis use problems and lower levels of non-standard cannabis use. Higher levels of conformity motives were significant and weakly linked to lower non-standard cannabis use and higher cannabis use problems. Coping motives were significantly and positively associated with non-standard cannabis use and cannabis use problems (with moderate and weak effect sizes, respectively).

Table 4 summarizes the total, direct and indirect effects between hostility and cannabis use outcomes in the trimmed mediation model. Hostility presented non-significant total and direct effects on non-standard cannabis use. Indirect effects between hostility and non-standard cannabis use were not estimated due to the non-significant total effect. Hostility presented significant total and direct effects on cannabis use problems. Additionally, four significant indirect pathways were identified in the trimmed mediation model. First, higher levels of hostility were associated with increased rates of AR which in turn had a positive effect on cannabis use problems. Second, higher levels of hostility predicted higher levels of coping motives which subsequently had a positive link with cannabis use problems. Third, hostility was positively related to elevated rates of AR which in turn predicted higher levels of conformity motives which in turn had a positive association with cannabis use problems. Fourth, hostility positively predicted AR which in turn was positively associated with coping motives which subsequently contributed to higher rates of cannabis use problems. These four indirect effects represented partial mediation effects due to the significant direct effect of hostility on cannabis use problems.

## 4 Discussion

The aim of the present study was to investigate the mediating role of AR and cannabis use motives on the relationship between hostility and problematic cannabis use. It was expected that the hypothesized mediation model can provide initial data on how the construct of AR can account for problematic cannabis use. To ease the interpretation, the findings of the trimmed mediation model is discussed in this section.

Predictor variables of the mediation model presented divergent associations with the two cannabis use outcomes, non-standard cannabis use and cannabis use problems. On one hand, a significant, positive direct effect of hostility on cannabis use problems was presented in the mediation model (over the effects of AR and cannabis motives). This relationship is in accordance with previous studies which demonstrated positive association between cannabis use and anger/hostility (Ansell et al., 2015, Pahl et al., 2011, Wycoff et al., 2018). AR also presented a significant and positive link with cannabis use problems. Although this was the first time that the associations between AR and cannabis use outcomes were investigated, previous findings also indicated positive correlations between problematic cannabis use and different forms of RNT (e.g., depressive rumination, post-event processing) (Adrian et al., 2014, Bravo et al., 2019, Ecker and Buckner, 2018). On the other hand, non-standard cannabis use had a non-significant relationship with hostility and a significant and negative relationship with AR. Non-standard cannabis use was defined by the frequencies of cannabis use before midday and when alone. Considering that high proportions of the sample were regular cannabis users, it might be possible that these two cannabis use characteristics were rather indirect indicators of frequent cannabis use (and not necessarily indicated problems and negative consequences due to use). That is, these findings might imply that hostility/anger and AR can show distinct associations with risky forms of cannabis use and problems associated with cannabis use. Some of the previous findings also reported weak or non-significant relationships between levels of cannabis use (e.g., frequency, quantity) and distressful affective symptoms (e.g., depression, anxiety, hostility), depressive rumination (Ansell et al., 2015, Bravo et al., 2019, Twomey, 2017). A similar pattern was demonstrated for the predictive effects of conformity motives: there were negative and positive associations with non-standard cannabis use and cannabis use problems, respectively. A meta-analysis also showed divergent associations of conformity motives with cannabis use and problems (Bresin & Mekawi, 2019). In line with the existing literature, coping motives were positively associated with both outcomes of problematic cannabis use (Bresin & Mekawi, 2019).

Four significant indirect effects were identified in the mediation model. The mediating effect of AR was presented in three significant indirect effects. The effect of hostility on cannabis use problems was significantly mediated (i) by AR, (ii) by AR and coping motives, and (iii) by AR and conformity motives. The single-mediation effect via AR shows similarities with those previous findings which demonstrated the mediating effect of other forms of RNT (e.g., brooding, post-event processing) on the associations between psychopathological symptoms (e.g., depression, social anxiety) and problematic cannabis use (Adrian et al., 2014, Ecker and Buckner, 2018). However, these studied did not control for the effects of cannabis use motives. In the mediation model of Bravo et al. (2019) – which simultaneously considered RNT and cannabis use motives in the context of depressive rumination – the single-mediation effects via depressive rumination were non-significant between depressive symptoms and problematic cannabis use. On the other hand, the double-mediation effect via AR and coping motives replicates Bravo et al.’s (2019) findings in the context of AR. In the latter study it was shown that the association between depressive symptoms and cannabis use problems was mediated by problem-focused thoughts and coping motives (Bravo et al., 2019). The assumptions of the ECM can provide theoretical basis for these two indirect effects. According to the ECM, there is a bidirectional association between psychopathological symptoms (e.g., anger/hostility) and rumination which can lead to dysregulated and impulsive behaviors (e.g., substance use) to cope with and divert attention from negative affective states and distressful cognitions (Kelley et al., 2021, Selby et al., 2008, Selby et al., 2009). However, due to the cross-sectional nature of the present study, cautious interpretation of these mechanisms is warranted.

The double-mediation effect via AR and conformity motives was unexpected based on previous literature. Similar to coping motives, negative reinforcement mechanisms describe conformity motives: individuals consume cannabis in order to avoid social rejection and disapproval (Cooper et al., 2015, Simons et al., 1998). It was assumed that substance use motivated by negative reinforcement mechanisms can lead to negative outcomes of substance use due to cognitive distortions, such as preference for interpreting negatively a neutral event, or not considering the long-term consequences of substance use if short-term benefits are attainable (Cooper et al., 2015). These underlying cognitive biases of conformity motives might show some similarities with the cognitive structure of AR which can explain the positive link between them. Moreover, previous studies also showed that both conformity motives and AR can be associated with higher rates of psychopathology. For example, it might be possible that the positive association between conformity motives and AR can be accounted for higher levels of general psychopathological severity, social anxiety and anxiety sensitivity (Cooper et al., 2015, Horváth et al., 2020). However, these suggested mechanisms should be interpreted cautiously as they were not tested in the present study. A positive link was also demonstrated between depressive rumination and conformity motives in the study of Bravo et al. (2019), which might suggest that using cannabis more frequently for conformity reasons might be associated with the preference for RNT. However, the indirect effect via these variables were non-significant in that study (Bravo et al., 2019).

Finally, an additional significant indirect effect in the mediation model indicated that hostility is positively associated with coping motives which subsequently contributed to more problematic cannabis use. It might be possible that individuals who lack the use of effective emotion regulation strategies might consume cannabis for self-medication purposes in response to the symptoms of anger/hostility and aggressive tendencies (Dillon et al., 2021). This significant indirect effect corresponds with previous findings which showed the mediating effect of coping motives on the relationship between distressful psychopathological symptoms (e.g., depression, social anxiety, obsessive-compulsivity) and problematic cannabis use (Bravo et al., 2019, Buckner et al., 2007, Spradlin et al., 2017).

It is important to note that enhancement, expansion and social motives were not included in the final, trimmed mediation model. These motivational dimensions did not show significant associations with hostility and AR – except for the negative link between hostility and expansion motives. Existing studies showed mixed and equivocal findings on the personality and psychopathological correlates of these three cannabis use motives (Cooper et al., 2015). A potential assumption for the (predominantly) non-significant associations can be that these positively reinforcing motives (i.e., using cannabis to reach a desirable and positive state) might be less tightly related to emotional or affective states (Cooper et al., 2015).

### 4.1 Limitations

Several methodological limitations should be considered regarding the present findings. First, the non-representative and convenience sample of the present study restricts the generalizability of the findings to the broader population of cannabis users (e.g., regular cannabis users were most likely over-represented in the sample). Second, the present analyses did not cover several theoretically and methodologically relevant variables. For example, in absence of other measures of RNT, it was not possible to completely assess the specific role of AR among cannabis users. By including other constructs of RNT in the mediation model (e.g., depressive rumination, self-critical rumination, worry), it would have been possible to examine the unique effect of AR on problematic cannabis use over the effects of other RNT constructs. Moreover, building on the assumptions of the multiple systems model of AR, the present study missed to investigate the cyclical association between executive functioning, self-control and AR, and their interactive effect on problematic cannabis use (Denson, 2013). Third, as the present study used a cross-sectional design, it was not possible to explore and specify the causal associations and bidirectional relationships between the variables (e.g., cannabis use can precede AR and hostility, and vice versa). Finally, several important statistical aspects were not considered during the analyses which might biased the findings. For example, the present study did not test for common method variance between the variables of the mediation model; did not test for measurement invariance of the main study variables in terms of gender and age; did not test for the influential effect of outlier cases; and it was also not possible to calculate robust, asymmetrical confidence intervals (e.g., by using bias-corrected and accelerated bootstrap method) for the indirect effects to have more reliable results. Low internal reliabilities of the two variables of cannabis use problems also should be considered as a limitation of the present study. A possible explanation for these results can be that these latent variables were defined by only two and four observed indicators.

## 5 Conclusions

The present study was the first that examined the construct of AR in the context of cannabis use. Multiple possible risk mechanisms via AR and cannabis use motives were suggested in the context of problematic cannabis use. Namely, a single-mediation effect via AR and two double-mediation effects via AR and coping motives and via AR and conformity motives were demonstrated between the positive association between hostility and cannabis use problems. Future research should consider using longitudinal research design (e.g., ecological momentary assessment) to explore the directional associations between the variables. Findings of the present study might have to be considered in the therapeutic programs that work with problematic cannabis users. For example, the use of mindfulness-based interventions was suggested for professionals working with individuals showing problematic substance use or anger disturbances (Chiesa and Serretti, 2014, Wright et al., 2009). Mindfulness-based interventions recommend applying a non-judgmental and detached observation and acceptance of the present emotions, body feelings, and cognitions (e.g., related to anger/hostility). This mindset might contribute to observe and interpret more precisely the antecedents, the development process, and consequences of anger/hostility as well as their potential role regarding cannabis use (e.g., recognizing distressful affective states and cues which might provoke craving for cannabis use). As a consequence of these changes, affected individuals might increase their ability for tolerating anger-provoking events and coping more efficiently with intense anger/hostile affective states (e.g., not using avoidance- or escape-based strategies, challenging rumination-based processes, teaching relaxation techniques in an intervention). Mindfulness-based interventions can also help to observe and accept the process of cannabis use motivated by negative reinforcement-based processes which aim to avoid distressful negative emotions (e.g., self-medication tendencies in response to anger/hostility), and to cope more efficiently with these negative affective states (Chiesa and Serretti, 2014, Wright et al., 2009). Forgiveness-based interventions can also be useful for individuals showing problems with substance use and handling anger/hostility (Goldman & Wade, 2012). For example, these interventions might focus on recalling the anger/hostility-provoking event and -related experiences, fostering to empathize in such situations, practicing and committing to forgiveness (Goldman & Wade, 2012).

## CRediT authorship contribution statement

**Zsolt Horváth:** Conceptualization, Methodology, Formal analysis, Data curation, Visualization, Writing – original draft, Writing – review & editing. **Gyöngyi Kökönyei:** Conceptualization, Methodology, Supervision, Writing – review & editing. **Péter Sárosi:** Methodology, Investigation, Writing – review & editing. **Mónika Koós:** Methodology, Investigation, Writing – review & editing. **Zsolt Demetrovics:** Conceptualization, Supervision, Writing – review & editing. **Róbert Urbán:** Conceptualization, Supervision, Writing – review & editing.

## Declaration of Competing Interest

The authors declare that they have no known competing financial interests or personal relationships that could have appeared to influence the work reported in this paper.
