# Supplementary material for: The mediating effect of anger rumination, coping and conformity motives on the association between hostility and problematic cannabis use
Source: Addict Behav Rep. 2022 Jul 9;16:100447. doi: 10.1016/j.abrep.2022.100447 (PMC9293590; doi:10.1016/j.abrep.2022.100447)
Supplement: Supplementary data 1 [file mmc1.docx]

Supplementary Table 1. The measurement model of hostility in the mediation model (one-factor model)

|  | Complete mediation model  λ | Trimmed mediation model  λ |
| --- | --- | --- |
| Easily annoyed | 0.76 | 0.76 |
| Temper outbursts | 0.79 | 0.80 |
| Urges to harm someone | 0.80 | 0.80 |
| Urges to break things | 0.76 | 0.76 |
| Arguing frequently | 0.63 | 0.63 |
| Internal consistency (McDonald’s ω) | 0.87 | 0.87 |

Note. λ: Standardized factor loadings. All factor loadings are significant at least p<0.001 level.

Supplementary Table 2. The bifactor model of anger rumination

|  | General anger rumination factor | Specific factors | | | |
| --- | --- | --- | --- | --- | --- |
|  |  | Angry after-thoughts (AA) | Angry memories (AM) | Thoughts of revenge (TR) | Under-standing causes (UC) |
| Standardized factor loadings (λ) | | | | | |
| 7. After an argument is over, I keep fighting with this person in my imagination | 0.67*** | 0.23*** |  |  |  |
| 8. Memories of being aggravated pop up into my mind before I fall asleep | 0.68*** | 0.01 |  |  |  |
| 9. Whenever I experience anger, I keep thinking about it for a while | 0.67*** | 0.20*** |  |  |  |
| 17. Memories of even minor annoyances bother me for a while | 0.76*** | 0.18*** |  |  |  |
| 18. When something makes me angry, I turn this matter over and over again in my mind | 0.76*** | 0.57*** |  |  |  |
| 19. I re-enact the anger episode in my mind after it has happened | 0.75*** | 0.54*** |  |  |  |
| 1. I ruminate about my past anger experiences | 0.70*** |  | 0.44*** |  |  |
| 2. I ponder about the injustices that have been done to me | 0.64*** |  | 0.20** |  |  |
| 3. I keep thinking about events that angered me for a long time | 0.79*** |  | 0.25*** |  |  |
| 5. I think about certain events from a long time ago and they still make me angry | 0.77*** |  | 0.26*** |  |  |
| 14. I feel angry about certain things in my life | 0.58*** |  | -0.02 |  |  |
| 4. I have long living fantasies of revenge after the conflict is over | 0.64*** |  |  | 0.63*** |  |
| 6. I have difficulty forgiving people who have hurt me | 0.53*** |  |  | 0.23*** |  |
| 13. I have day dreams and fantasies of violent nature | 0.56*** |  |  | 0.29*** |  |
| 15. When someone makes me angry, I can't stop thinking about how to get back at this person | 0.60*** |  |  | 0.67*** |  |
| 10. I have had times when I could not stop being preoccupied with a particular conflict | 0.69*** |  |  |  | 0.09 |
| 11. I analyze events that make me angry | 0.41*** |  |  |  | 0.18* |
| 12. I think about the reasons people treat me badly | 0.62*** |  |  |  | 0.77** |
| 16. When someone provokes me, I keep wondering why this should have happened to me | 0.51*** |  |  |  | 0.31** |
| Reliability indices | | | | | |
| Explained common variance (ECV) | 74% | 7% | 3% | 9% | 7% |
| Omega (ω) | 0.95 | 0.91 | 0.86 | 0.84 | 0.77 |
| Hierarchical omega (ωH) | 0.90 | 0.13 | 0.08 | 0.32 | 0.21 |
| Relative omega (ωH/ω) | 94% | 14% | 10% | 38% | 27% |
| H-index | 0.94 | 0.50 | 0.30 | 0.61 | 0.62 |
| Factor determinacy index (FDI) | 0.97 | 0.71 | 0.54 | 0.78 | 0.79 |
| Percentage of uncontaminated correlations (PUC) | 0.78 | - | - | - | - |

Note. Level of significance: *p<0.050; **p<0.010; ***p<0.001.

Supplementary Table 3. The measurement model of anger rumination in the mediation model (one-factor model with residual covariances)

|  | Complete mediation model | Trimmed mediation model |
| --- | --- | --- |
| Standardized factor loadings (λ) | | |
| 1. I ruminate about my past anger experiences | 0.68 | 0.68 |
| 2. I ponder about the injustices that have been done to me | 0.63 | 0.63 |
| 3. I keep thinking about events that angered me for a long time | 0.80 | 0.81 |
| 4. I have long living fantasies of revenge after the conflict is over | 0.63 | 0.64 |
| 5. I think about certain events from a long time ago and they still make me angry | 0.77 | 0.77 |
| 6. I have difficulty forgiving people who have hurt me | 0.52 | 0.52 |
| 7. After an argument is over, I keep fighting with this person in my imagination | 0.65 | 0.65 |
| 8. Memories of being aggravated pop up into my mind before I fall asleep | 0.69 | 0.68 |
| 9. Whenever I experience anger, I keep thinking about it for a while | 0.66 | 0.65 |
| 10. I have had times when I could not stop being preoccupied with a particular conflict | 0.68 | 0.68 |
| 11. I analyze events that make me angry | 0.37 | 0.37 |
| 12. I think about the reasons people treat me badly | 0.62 | 0.62 |
| 13. I have day dreams and fantasies of violent nature | 0.63 | 0.62 |
| 14. I feel angry about certain things in my life | 0.59 | 0.60 |
| 15. When someone makes me angry, I can't stop thinking about how to get back at this person | 0.61 | 0.61 |
| 16. When someone provokes me, I keep wondering why this should have happened to me | 0.53 | 0.53 |
| 17. Memories of even minor annoyances bother me for a while | 0.77 | 0.77 |
| 18. When something makes me angry, I turn this matter over and over again in my mind | 0.75 | 0.75 |
| 19. I re-enact the anger episode in my mind after it has happened | 0.73 | 0.73 |
| Internal consistency (McDonald’s ω) | 0.93 | 0.93 |
| Error correlations (r) - Angry after-thoughts (AA)-specific items | | |
| Item 7 – Item 8 | 0.03 | 0.04 |
| Item 7 – Item 9 | 0.26*** | 0.27*** |
| Item 7 – Item 17 | 0.10 | 0.11 |
| Item 7 – Item 18 | 0.26*** | 0.27*** |
| Item 7 – Item 19 | 0.27*** | 0.28*** |
| Item 8 – Item 9 | 0.10* | 0.11* |
| Item 8 – Item 17 | -0.06 | -0.05 |
| Item 8 – Item 18 | -0.06 | -0.05 |
| Item 8 – Item 19 | 0.03 | 0.04 |
| Item 9 – Item 17 | -0.06 | -0.05 |
| Item 9 – Item 18 | 0.20*** | 0.21*** |
| Item 9 – Item 19 | 0.26*** | 0.26*** |
| Item 17 – Item 18 | 0.31*** | 0.31*** |
| Item 17 – Item 19 | 0.21*** | 0.21*** |
| Item 18 – Item 19 | 0.73*** | 0.73*** |
| Error correlations (r) - Angry memories (AM)-specific items | | |
| Item 1 – Item 2 | 0.16** | 0.15** |
| Item 1 – Item 3 | 0.22*** | 0.22*** |
| Item 1 – Item 5 | 0.30*** | 0.29*** |
| Item 1 – Item 14 | 0.00 | 0.00 |
| Item 2 – Item 3 | 0.21*** | 0.21*** |
| Item 2 – Item 5 | 0.04 | 0.04 |
| Item 2 – Item 14 | -0.01 | -0.01 |
| Item 3 – Item 5 | 0.09 | 0.08 |
| Item 3 – Item 14 | -0.08 | -0.09 |
| Item 5 – Item 14 | -0.07 | -0.07 |
| Error correlations (r) - Thoughts of revenge (TR)-specific items | | |
| Item 4 – Item 6 | 0.24*** | 0.23*** |
| Item 4 – Item 13 | 0.24** | 0.24** |
| Item 4 – Item 15 | 0.67*** | 0.67*** |
| Item 6 – Item 13 | 0.04 | 0.04 |
| Item 6 – Item 15 | 0.24*** | 0.24*** |
| Item 13 – Item 15 | 0.24** | 0.24** |
| Error correlations (r) - Under-standing causes (UC)-specific items | | |
| Item 10 – Item 11 | 0.26*** | 0.25*** |
| Item 10 – Item 12 | 0.11* | 0.11* |
| Item 10 – Item 16 | -0.08 | -0.08 |
| Item 11 – Item 12 | 0.21*** | 0.21*** |
| Item 11 – Item 16 | 0.06 | 0.06 |
| Item 12 – Item 16 | 0.35*** | 0.36*** |

Note. All factor loadings are significant at least p<0.001 level. Level of significance for the error correlations: *p<0.050; **p<0.010; ***p<0.001.

Supplementary Table 4. The measurement model of cannabis use motives in the mediation model (correlated five-factor model)

|  | Complete mediation model | Trimmed mediation model |
| --- | --- | --- |
| Standardized factor loadings (λ) – Conformity motives | | |
| Because my friends pressure me to use marijuana | 0.53 | 0.55 |
| So that others won’t kid me about not using marijuana | 0.65 | 0.62 |
| To fit in with the group I like | 0.81 | 0.78 |
| To be liked | 0.87 | 0.86 |
| So I won’t feel left out | 0.85 | 0.89 |
| Internal consistency (McDonald’s ω) | 0.86 | 0.86 |
| Standardized factor loadings (λ) – Coping motives | | |
| To forget my worries | 0.78 | 0.86 |
| Because it helps me when I feel depressed or nervous | 0.83 | 0.82 |
| To cheer me up when I am in a bad mood | 0.86 | 0.82 |
| To forget about my problems | 0.67 | 0.48 |
| Because I feel more self-confident and sure of myself | 0.85 | 0.87 |
| Internal consistency (McDonald’s ω) | 0.90 | 0.88 |
| Standardized factor loadings (λ) – Enhancement motives | | |
| Because I like the feeling | 0.74 | - |
| Because it’s exciting | 0.59 | - |
| To get high | 0.81 | - |
| Because it gives me a pleasant feeling | 0.76 | - |
| Because it’s fun | 0.60 | - |
| Internal consistency (McDonald’s ω) | 0.83 | - |
| Standardized factor loadings (λ) – Expansion motives | | |
| To know myself better | 0.76 | - |
| Because it helps me be more creative and original | 0.83 | - |
| To understand things differently | 0.93 | - |
| To expand my awareness | 0.92 | - |
| To be more open to experiences | 0.83 | - |
| Internal consistency (McDonald’s ω) | 0.93 | - |
| Standardized factor loadings (λ) – Social motives | | |
| Because it helps me enjoy a party | 0.74 | - |
| To be sociable | 0.68 | - |
| Because it makes social gatherings more fun | 0.84 | - |
| Because it improves parties and celebrations | 0.90 | - |
| To celebrate a special occasion with friends | 0.70 | - |
| Internal consistency (McDonald’s ω) | 0.88 | - |
| Correlations between the latent factors (r) | | |
| Conformity motives – Coping motives | 0.18** | 0.18** |
| Conformity motives – Enhancement motives | 0.14* | - |
| Conformity motives – Expansion motives | 0.04 | - |
| Conformity motives – Social motives | 0.43*** | - |
| Coping motives – Enhancement motives | 0.48*** | - |
| Coping motives – Expansion motives | 0.30*** | - |
| Coping motives – Social motives | 0.40*** | - |
| Enhancement motives – Expansion motives | 0.35*** | - |
| Enhancement motives – Social motives | 0.60*** | - |
| Expansion motives – Social motives | 0.36*** | - |

Note. All factor loadings are significant at least p<0.001 level. Level of significance for the correlations between the latent factors: *p<0.050; **p<0.010; ***p<0.001.

Supplementary Table 5. The measurement model of harmful cannabis use in the mediation model (correlated two-factor model)

|  | Complete mediation model | Trimmed mediation model |
| --- | --- | --- |
| Standardized factor loadings (λ) – Non-standard cannabis use | | |
| Cannabis use before midday | 0.73 | 0.65 |
| Cannabis use when alone | 0.47 | 0.53 |
| Internal consistency (McDonald’s ω) | 0.53 | 0.52 |
| Standardized factor loadings (λ) – Cannabis use problems | | |
| Memory problems | 0.53 | 0.53 |
| Being encouraged to reduce or stop cannabis use by friends or family | 0.54 | 0.51 |
| Unsuccessful attempts to reduce or stop cannabis use | 0.46 | 0.48 |
| Problems because of cannabis use | 0.65 | 0.67 |
| Internal consistency (McDonald’s ω) | 0.63 | 0.63 |
| Correlation between the two factors (r) | 0.29 | 0.29 |

Note. All factor loadings and correlations are significant at least p<0.001 level.
